# Very Long O-antigen Chains Enhance Fitness during *Salmonella*-induced Colitis by Increasing Bile Resistance

**DOI:** 10.1371/journal.ppat.1002918

**Published:** 2012-09-20

**Authors:** Robert W. Crawford, A. Marijke Keestra, Sebastian E. Winter, Mariana N. Xavier, Renée M. Tsolis, Vladimir Tolstikov, Andreas J. Bäumler

**Affiliations:** 1 Department of Medical Microbiology and Immunology, School of Medicine, University of California at Davis, Davis, California, United States of America; 2 Metabolomics Core Laboratory, Genome Center, University of California at Davis, Davis, California, United States of America; Faculté de Médecine Paris Descartes, site Necker, France

## Abstract

Intestinal inflammation changes the luminal habitat for microbes through mechanisms that have not been fully resolved. We noticed that the FepE regulator of very long O-antigen chain assembly in the enteric pathogen *Salmonella enterica* serotype Typhimurium (*S.* Typhimurium) conferred a luminal fitness advantage in the mouse colitis model. However, a *fepE* mutant was not defective for survival in tissue, resistance to complement or resistance to polymyxin B. We performed metabolite profiling to identify changes in the luminal habitat that accompany *S.* Typhimurium-induced colitis. This analysis suggested that *S.* Typhimurium-induced colitis increased the luminal concentrations of total bile acids. A mutation in *fepE* significantly reduced the minimal inhibitory concentration (MIC) of *S.* Typhimurium for bile acids *in vitro*. Oral administration of the bile acid sequestrant cholestyramine resin lowered the concentrations of total bile acids in colon contents during *S.* Typhimurium infection and significantly reduced the luminal fitness advantage conferred by the *fepE* gene in the mouse colitis model. Collectively, these data suggested that very long O-antigen chains function in bile acid resistance of *S.* Typhimurium, a property conferring a fitness advantage during luminal growth in the inflamed intestine.

## Author Summary

Microbial communities inhabiting the intestinal tract have been characterized using high-throughput sequencing approaches, but little is known about factors that change their luminal habitat. Using metabolite profiling we discovered that luminal concentrations of total bile acids become significantly elevated during *Salmonella*-induced colitis. Very long O-antigen chains of *Salmonella enterica* serotype Typhimurium (*S.* Typhimurium) were required for luminal growth in the presence of such elevated bile concentrations during colitis. In contrast, very long O-antigen chains were dispensable for luminal growth in the presence of normal concentrations of bile acids or for growth in organs of mice. Thus, elevated bile concentrations during *Salmonella*-induced colitis change the luminal habitat and microbial growth in this environment requires increased bile resistance, which in case of *S.* Typhimurium is conferred by very long O-antigen chains.

## Introduction

*Salmonella enterica* serotype Typhimurium (*S.* Typhimurium) is an important cause of human gastroenteritis [1]. Upon ingestion, a fraction of the *S.* Typhimurium population enters intestinal epithelial cells using the invasion-associated type III secretion system (T3SS-1) [2], which is followed by macrophage survival mediated by a second type III secretion system (T3SS-2) [3]. The deployment of T3SS-1 and T3SS-2 triggers acute intestinal inflammation [4] and the resulting changes in the environment enhance growth of the luminal fraction of the *S.* Typhimurium population [5], [6], [7], [8] despite the fact that concentrations of antimicrobial substances are elevated in this habitat [9], [10], [11].

The surface of *S.* Typhimurium, which is exposed to antimicrobial substances in the inflamed gut, is decorated with lipopolysaccharide (LPS). The oligosaccharide core connects the lipid A moiety, which anchors the LPS molecule in the outer membrane, to O-antigen repeat units that extend from the bacterial surface. Each O-antigen repeat unit is composed of a trisaccharide backbone, consisting of α-D-mannose-(1,4)-α-L-rhamnose-(1,3)-α-D-galactose, and a branching sugar (abequose) that is α-(1,3)-linked to D-mannose in the backbone. *S.* Typhimurium can produce short LPS species containing between 1 and 15 O-antigen repeat units. Additionally, *S.* Typhimurium synthesizes LPS species containing a greater number of O antigen repeat units, which requires the length regulators WzzB [12], [13] and FepE [14]. The WzzB protein regulates the assembly of long LPS species carrying between 16 and 35 O-antigen repeat units [15] while the FepE protein controls the biosynthesis of very long LPS species with more than 100 O-antigen repeat units [14]. These regulatory mechanisms give rise to a tri-modal distribution in LPS length.

There is general agreement that WzzB-dependent assembly of long O-antigen chains (16–35 repeat units) provides a fitness advantage by conferring resistance to complement [14], [16]. In contrast, the function of very long O-antigen chains (>100 repeat units) during host microbe interaction remains unclear. The absence of very long O-antigen species in a *fepE* mutant reduces *in vitro* translocation of SipA [16], a T3SS-1 effector protein that significantly contributes to the induction of inflammation in the mouse colitis model [17]. However, *in vivo* the O-antigen is not required for T3SS-1-dependent induction of intestinal inflammatory responses [18]. These data indicate that SipA translocation proceeds through an O-antigen-independent pathway *in vivo*. One study suggests that very long O-antigen inhibits macrophage phagocytosis [19], while others observed no inhibition [16]. A *fepE* mutant and its *S.* Typhimurium wild-type parent are equally resistant to complement *in vitro*, although a *wzzB fepE* mutant is more serum sensitive than a *wzzB* mutant [14], [16], [20]. Importantly, an *S.* Typhimurium *fepE* mutant retains full virulence in the mouse typhoid model [14], suggesting that complement resistance and resistance to phagocyte-mediated killing mechanisms are independent of very long O-antigen chains *in vivo*. Since no phenotype has been described for a *S.* Typhimurium *fepE* mutant in mouse models, it remains unclear which selective forces prevent loss of very long O-antigen chains.

In the absence of selective forces to maintain the costly production of very long O-antigen chains, the *fepE* gene is predicted to accumulate point mutations and eventually become inactive. Interestingly, *fepE* is a pseudogene in *S. enterica* serotype Typhi, the causative agent of typhoid fever [21]. We thus reasoned that *fepE* might be involved in aspects of host pathogen interaction that are important during gastroenteritis (caused by *S.* Typhimurium) but dispensable during typhoid fever (caused by *S.* Typhi). While *S.* Typhimurium transmission requires maximum growth in the lumen of the inflamed intestine [22], *S.* Typhi persists in the human population through chronic gall bladder carriage [23], [24]. Based on these considerations, we proposed that very long O-antigen chains might enhance the fitness of *S.* Typhimurium in the environment of the inflamed intestine during gastroenteritis. We tested this hypothesis using the mouse colitis model and elucidated the underlying mechanism.

## Results

### Very long O-antigen chains confer a luminal growth advantage in the inflamed gut

To study the role of very long O-antigen chains, we generated a *fepE* mutant of the *S.* Typhimurium wild type strain IR715. The *fepE* mutant (RC31) was deficient for producing very long O-antigen chains, and this defect could be restored by introducing the cloned *fepE* gene (pCR37) on a plasmid (Fig. S1A). Consistent with previous reports [14], [16], the *S.* Typhimurium wild type (IR715) and the *fepE* mutant (RC31) exhibited similar levels of serum resistance (Fig. S1B). In contrast, a *wzzB* mutant that lacked long O-antigen chains but retained the ability to produce very long O-antigen chains (Fig. S1A) exhibited significantly (*P*<0.01) reduced serum resistance (Fig. S1B).

Genetically resistant mice infected with *S.* Typhimurium exhibit little intestinal inflammation during the first three days after inoculation (mouse typhoid model) [25]. In contrast, mice treated with streptomycin develop severe acute cecal inflammation within 2 days after infection (mouse colitis model). To determine whether very long O-antigen chains confer a fitness advantage during conditions of acute intestinal inflammation, we performed a pilot experiment in which streptomycin pre-treated, genetically susceptible mice (C57BL/6J) were infected intragastrically with an equal mixture of the *S.* Typhimurium wild type (IR715) and a *fepE* mutant (RC31) (mouse colitis model). By day four after infection, mice became moribund and the wild type was recovered in higher numbers than the *fepE* mutant from intestinal contents, but not from liver and spleen (Fig. S2).

**Figure 1:**
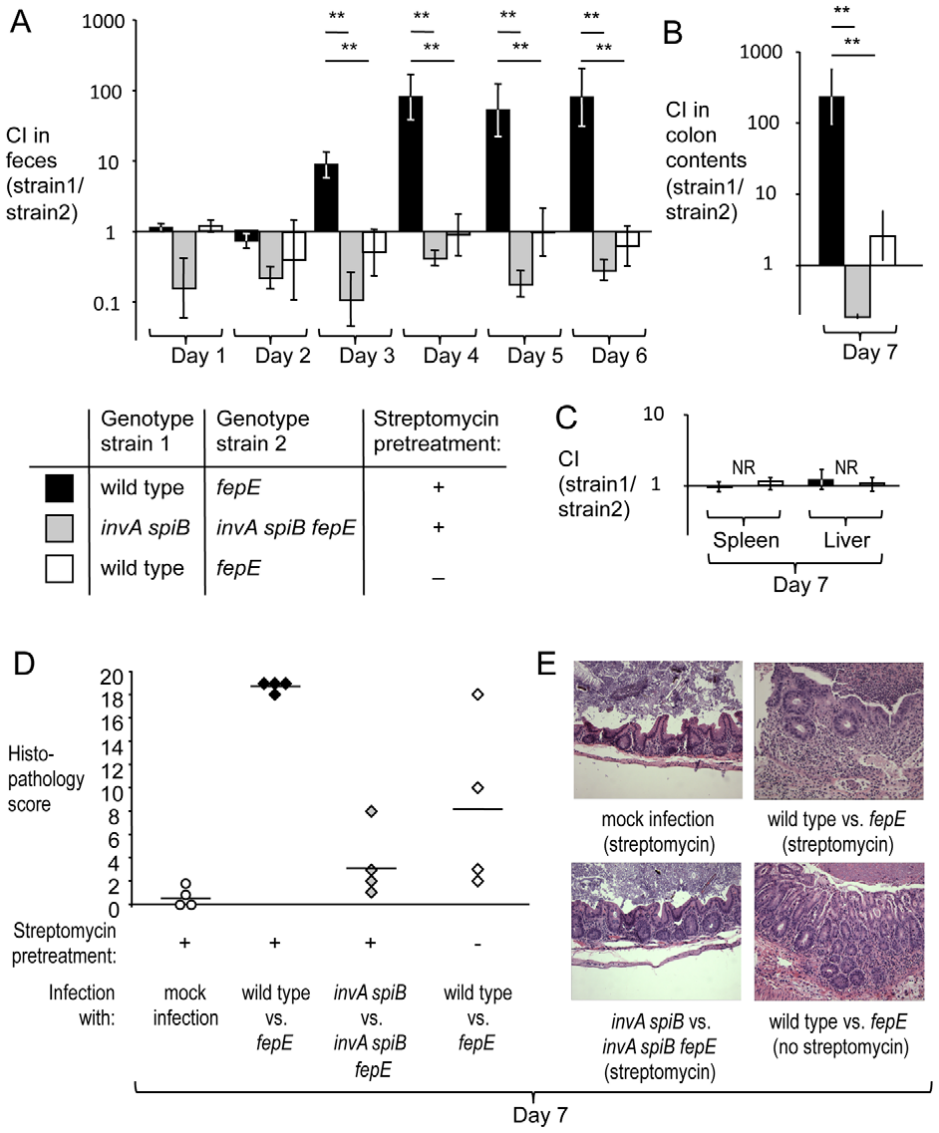
Very long O-antigen chains confer a fitness advantage in the lumen of the inflamed gut. Mice (129/SvJ) were treated with streptomycin or were left untreated and were subsequently infected with equal mixtures of the indicated *S.* Typhimurium strains. (A) Competitive indices (CI) recovered from feces over time. (B) Competitive indices recovered from colon contents. (C) Competitive indices recovered from the liver and spleen. (A–C) Bars represent geometric means ± standard error. *, *P*<0.05; **, *P*<0.01, NR, no bacteria recovered. (D) Combined histopathology score of pathological changes observed in sections from the cecum. Lines represent averages. Each circle or diamond represents the combined histopathology score from an individual animal. (E) Representative images of histopathological changes.

To determine whether the luminal fitness advantage conferred by the *fepE* gene would increase over time, groups of streptomycin pre-treated, genetically resistant mice (129/SvJ) were infected intragastrically with an equal mixture of the *S.* Typhimurium wild type (IR715) and a *fepE* mutant (RC31) (mouse colitis model). For comparison, mice (129/SvJ) that had not been pre-treated with streptomycin were infected with the same mixture of *S.* Typhimurium strains (mouse typhoid model). In the mouse typhoid model, the wild type did not exhibit a luminal growth advantage over the *fepE* mutant as indicated by recovery of bacteria from feces (Fig. 1A) or colon contents (Fig. 1B). In contrast, the wild type was recovered in significantly higher numbers (*P*<0.05) from feces starting at day 3 after infection in the mouse colitis model (Fig. 1A). By day 7 after infection, the wild type was recovered in greater than 100-fold excess over the *fepE* mutant (Fig. 1B). In both the mouse typhoid model and in the mouse colitis model, the *S.* Typhimurium wild type and *fepE* mutant were recovered in similar numbers from the liver and spleen (Fig. 1C), suggesting that very long O-antigen chains are dispensable for growth in tissue. As expected, histopathogical analysis revealed a greater severity of cecal inflammation in the mouse colitis model compared to the mouse typhoid model seven days after infection (Fig. 1 and E). Bacteria recovered from intestinal contents of streptomycin pretreated mice exhibited strong expression of very long O-antigen chains, as indicated by Western blot analysis (Fig. S3).

Production of very long O-antigen chains had a similar effect on bacterial numbers in the mouse colitis model when we used CBA/J mice, a lineage that is also genetically resistant to *S.* Typhimurium infection (Fig. S4A and S4B). To assess complementation *in vivo*, streptomycin pre-treated CBA/J mice were infected with an equal mixture of a *S.* Typhimurium *fepE* mutant (RC61) and a derivative carrying the *fepE* gene inserted upstream of the *phoN* gene (*phoN*::*fepE*) (RC62). The complemented strain (RC62) was recovered in higher numbers from feces and colon contents (Fig. S4C), suggesting that an intact chromosomal copy of the *fepE* gene could restore the fitness defect of a *fepE* mutant. Next, we investigated whether the fitness advantage conferred by very long O-antigen chains was apparent in mice infected with individual *S.* Typhimurium strains (single infection design). Streptomycin pre-treated CBA/J mice were infected with either the *S.* Typhimurium wild type (IR715) or a *fepE* mutant (RC31) (mouse colitis model). Mice infected with the *S.* Typhimurium wild type shed significantly (*P*<0.01) higher bacterial numbers with their feces than mice infected with the *fepE* mutant (Fig. S4E), while similar bacterial loads were recovered from liver and spleen (Fig. S4D).

T3SS-1 and T3SS-2 cooperate to induce intestinal inflammation in the mouse colitis model [26], [27]. Inactivation of both T3SS-1 (through a mutation in *invA*) and T3SS-2 (through a mutation in *spiB*) renders the resulting *S.* Typhimurium double mutant strain unable to trigger intestinal inflammation [9]. To further investigate why very long O-antigen chains conferred a fitness advantage in the mouse colitis model, streptomycin pre-treated mice (129/SvJ) were infected with an equal mixture of an *invA spiB* mutant (SPN452) and an *invA spiB fepE* mutant (RC55). Mice infected with this mixture did not developed intestinal inflammation (Fig. 1A and 1B) and the *invA spiB* mutant did not exhibit a luminal growth advantage over the *invA spiB fepE* mutant (Fig. 1A and 1B). Due to the attenuation caused by mutations in *invA* and *spiB*, neither strain was recovered from the liver and spleen of mice seven days after infection (Fig. 1C). Collectively, these data pointed to the lumen of the acutely inflamed intestine as the habitat conferring a fitness advantage to *S.* Typhimurium expressing very long O-antigen chains. However, the mechanism by which very long O-antigen chains would provide a benefit in the environment of the inflamed gut remained unclear.

### Metabolite profiling of luminal changes during *S.* Typhimurium induced colitis

To identify potential small molecule candidates that might explain the fitness advantage conferred by very long O-antigen chains in the inflamed gut, groups of streptomycin pre-treated mice (n = 4) were inoculated with sterile LB broth (mock infection) or *S.* Typhimurium. Four days after infection, the ceca were collected and metabolites extracted from the mucosa with water. Metabolites in these cecal washes were analyzed after removing bacteria by filtration to avoid contamination of samples with bacterial intracellular metabolites. Samples underwent hydrophilic interaction chromatography - liquid chromatography/mass spectrometry (HILIC-LC/MS) metabolic profiling. Peak metabolite values were measured for cecal washes of each animal and data were transformed logarithmically (log_2_) for statistical analysis and determination of false discovery rates. More than 800 components were detected by HILIC-LC/MS. Principle component analysis revealed group clustering of samples from mock-infected mice and *S.* Typhimurium-infected mice (Fig. S5), suggesting that *S.* Typhimurium infection was accompanied by characteristic changes in the luminal environment.

**Figure 2:**
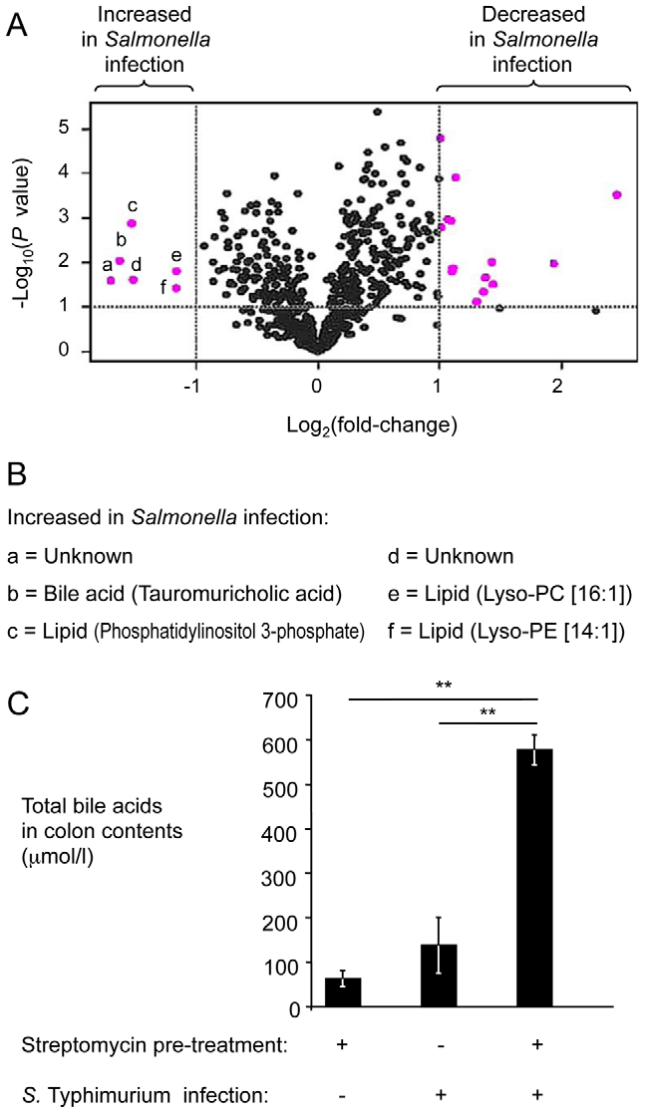
Bile acids are elevated during *S.* Typhimurium-induced colitis. (A) Groups of streptomycin pre-treated mice were inoculated with sterile LB broth (mock infection, n = 4) or with *S.* Typhimurium (n = 4). Four days after infection, water extracts of metabolites from the cecal mucosa were analyzed by HILIC-LC/MS metabolite profiling. For each component detected by HILIC-LC/MS, peak areas from four mock-infected animals were averaged and compared to the average peak areas from four *S.* Typhimurium-infected animals. Each dot in the volcano plot represents the fold-change between mock-infected and *S.* Typhimurium-infected mice for one component. Components with at least a 2-fold change and a *P* value of less than 0.1 are indicated in pink. (B) List of metabolites showing at least a 2-fold increase during *S.* Typhimurium infection. Letters correspond to letters in panel A. Lys-PE, lysophosphatidylethanolamine; Lys-PC, lysophosphatidylcholine. (C) Determination of concentrations of total bile acids in colon contents. Bars represent geometric means ± standard error. **, *P*<0.01.

We were able to assign metabolite identities to 67 components (Table S1). A total of 20 components met the criteria for at least a 2-fold change between samples from mock-infected and *S.* Typhimurium-infected mice and a *P* value of less than 0.1, which is illustrated in a volcano plot in Figure 2A. Of these, 15 components were considered significantly changed (*P*<0.05; *q*<0.1) between both groups. We were able to assign metabolite identities to four of the six components that were significantly (*P*<0.05; *q*<0.1) increased in samples from *S.* Typhimurium-infected mice compared to mock-infected animals (Fig. 2B). Interestingly, one of these metabolites, phosphatidylinositol 3-phosphate, is produced in host cells by the T3SS-1 effector protein SopB [28]. Two metabolites were breakdown products of phosphatidylethanolamine (lysophosphatidylethanolamine [14∶1]) and phosphatidylcholine (lysophosphatidylcholine [16∶1]), respectively. The fourth metabolite with significantly increased abundance (*P*<0.05; *q*<0.1) in samples from *S.* Typhimurium infected mice was the bile acid tauromurocholate. These data indicated that increased levels of bile acids might accompany *S.* Typhimurium infection, thereby identifying a potential candidate for an anti-microbial metabolite that is elevated during colitis.

To validate results from metabolite profiling, the concentration of total bile acids was determined in colon contents recovered from the mouse typhoid model and from the mouse colitis model using an enzymatic assay. The concentration of total bile acids was significantly (*P*<0.01) increased in the mouse colitis model, compared to the mouse typhoid model or to mock-infected mice (Fig. 2C). These data suggested that the concentration of total bile acids was increased during *S.* Typhimurium-induced colitis. Furthermore, these data established an inverse correlation between luminal concentrations of total bile acids (Fig. 2C) and the luminal fitness of the *fepE* mutant (Fig. 1A and 1B) but fell short of establishing cause and effect.

### Very long O-antigen chains increasing resistance to bile

To investigate whether an increased concentration of total bile acids during *S.* Typhimurium-induced colitis was the mechanism responsible for the fitness advantage conferred by very long O-antigen chains (Fig. 1), we first determined the minimal inhibitory concentration (MIC) of *S.* Typhimurium strains to ox bile extract (sodium choleate) *in vitro* (Table 1). The MIC of sodium choleate was identical for the *S.* Typhimurium wild type (IR715) and a *wzzB* mutant (RC46). However, compared to the wild type, the MIC of sodium choleate for the *fepE* mutant (RC31) was reduced. Introduction of the cloned *fepE* gene (pCR37) into the *fepE* mutant restored resistance to sodium choleate. Lack of very long O-antigen chains in the *fepE* mutant did not reduce the MIC for the anionic detergent sodium dodecyl sulfate (SDS) or for the cationic antimicrobial peptide polymyxin B. In contrast, a mutation in *pmrE*, a gene necessary for 4-aminoarabinose lipid A modification [29], increased sensitivity to the antimicrobial peptide polymyxin B, but not to the anionic detergent SDS. Collectively, these data suggested that very long O-antigen chains, but not long O-antigen chains, are specifically required for full resistance to bile acids.

**Table 1:** Very long O-antigen chains confer resistance to bile acids.

Genotype	MIC for Polymyxin B	MIC for SDS	MIC for sodium choleate	MIC for sodium choleate in the presence of 100 µg/ml cholestyramine resin
Wild-type	1.2 µg/ml	12%	12%	20%
*fepE*	1.2 µg/ml	12%	2%	20%
*fepE* (pCR37)	1.2 µg/ml	12%	12%	20%
*wzzB*	1.2 µg/ml	12%	12%	20%
*pmrE*	0.5 µg/ml	12%	12%	20%

Since the MIC of the *fepE* mutant for sodium choleate (2%) was nearly 100-fold higher than the concentration of bile acids measured in intestinal contents (Fig. 2C), we compared growth of the *S.* Typhimurium wild type and a *fepE* mutant in medium containing different concentrations of bile acids. As expected, medium containing 3% sodium choleate supported growth of the *S.* Typhimurium wild type (MIC 12%) but fully suppressed growth of the *fepE* mutant (MIC 2%) (Fig. S6A). Growth of the *fepE* mutant was retarded compared to that of the *S.* Typhimurium wild type in medium containing 0.3% sodium choleate (Fig. S6B) or 0.03% sodium choleate (Fig. S6C). Finally, both strains grew equally in medium containing 0.003% sodium choleate (Fig. S6D). These data suggested that very long O-antigen chains conferred a fitness advantage at concentrations of bile acids that were approximately two orders of magnitude below the MIC of the *fepE* mutant (Table 1).

The concentration of total bile acids in intestinal contents can be lowered by bile sequestrants, such as cholestyramine resin, a compound used clinically to restrict cholesterol and fat intake. To investigate whether bile sequestrants could rescue growth of the *fepE* mutant in medium containing sodium choleate, MIC values were determined *in vitro* in the presence of cholestyramine resin (Table 1). The *S.* Typhimurium wild type and the *fepE* mutant exhibited identical MIC values for sodium choleate in the presence of cholestyramine resin.

**Figure 3:**
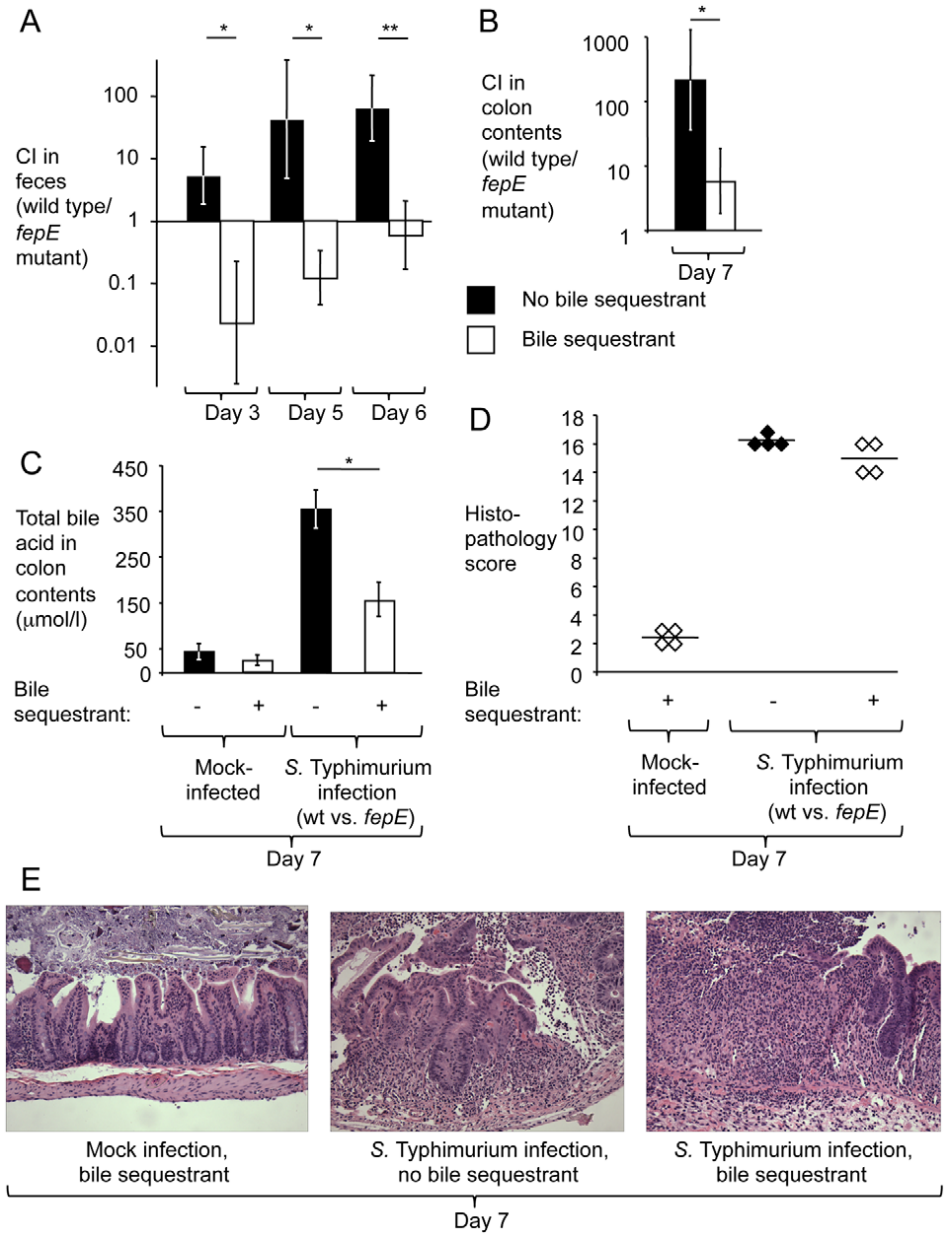
The bile sequestrant cholestyramine resin reduces the fitness advantage conferred by very long O-antigen chains in the mouse colitis model. Mice (129/SvJ) receiving chow containing cholestyramine resin or control chow were pre-treated with streptomycin and infected with an equal mixture of the *S.* Typhimurium wild type and a *fepE* mutant. (A) Competitive indices recovered from feces over time. (B) Competitive indices recovered from colon contents. (C) Determination of concentrations of total bile acids in colon contents. (A–C) Bars represent geometric means ± standard error. *, *P*<0.05; **, *P*<0.01. (D) Combined histopathology score of pathological changes observed in sections from the cecum. Lines represent averages. Each circle or diamond represents the combined histopathology score from an individual animal. (E) Representative images of histopathological changes.

To test whether increased concentrations of total bile acids are responsible for the fitness advantage conferred by very long O-antigen chains in the mouse colitis model, animals fed rodent chow containing cholestyramine resin or control chow were pretreated with streptomycin and inoculated with sterile LB broth (mock infection) or with an equal mixture of the *S.* Typhimurium wild type (IR715) and a *fepE* mutant (RC31). While the *S.* Typhimurium wild type was recovered in significantly higher numbers from feces (Fig. 3A) and colon contents (Fig. 3B) of mice fed control chow, this competitive advantage was abrogated in mice fed chow containing cholestyramine resin. Concomitantly, diet containing cholestyramine resin significantly (*P*<0.05) reduced the concentration of total bile acids in colon contents present during *S.* Typhimurium infection (Fig. 3C) but did not reduce the severity of intestinal inflammation (Fig. 3D and 3E). These data provided direct support for the idea that increased concentrations of total bile acids during *S.* Typhimurium-induced colitis reduced the fitness of bacteria lacking very long O-antigen chains.

## Discussion

*S.* Typhimurium-induced colitis is accompanied by elevated luminal concentrations of several antimicrobial proteins, including lipocalin-2 [9], calprotectin [11], regenerating islet-derived 3 gamma (RegIIIγ) [30] and RegIIIβ [10]. In some cases, specific resistance mechanisms against these antimicrobial proteins have been shown to confer a fitness advantage in the lumen of the inflamed gut. For example, the ability to synthesize the siderophore salmochelin confers resistance to lipocalin-2 [31], a property that boosts luminal growth of *S.* Typhimurium in the mouse colitis model [9]. Similarly, the high affinity zinc transporter ZnuABC confers resistance to the zinc-binding protein calprotectin, thereby enhancing luminal growth of *S.* Typhimurium during colitis [11].

Here we used metabolite profiling to determine whether small antimicrobial metabolites are elevated during *S.* Typhimurium-induced colitis. Our results identify bile acids as a class of small antimicrobial molecules whose concentration is elevated in the mouse colitis model. A previous metabolite profiling study detected elevated bile acid biosynthesis in the mouse typhoid model by analyzing acetonitrile extracts from feces [32]. Total concentrations of bile acids in feces and fecal water are elevated two to five times in patients with inflammatory bowel disease [33]. However, fecal concentrations of total bile acids are significantly decreased during dextran sulfate sodium (DSS)-induced colitis in rats [34]. Thus, an increased concentration of bile acids is not likely to be a general feature of intestinal inflammation, but might involve mechanisms that are only triggered in a subset of inflammatory disorders, including *S.* Typhimurium-induced colitis. It should be noted that due to absorption in the large intestine, bile concentrations might be higher in the ileum, the primary site of *S.* Typhimurium infection in humans. The mouse is not an ideal model to study luminal growth in the ileum, because the bulk of luminal *S.* Typhimurium are present in the cecum.

The absence of acute colitis during typhoid fever might explain why *fepE* is a pseudogene in *S.* Typhi [21]. However, *S.* Typhi requires bile resistance for persistence in the gall bladder. It is possible that other long surface carbohydrates, such as the Vi-capsular polysaccharide or the O-antigen capsule [35], [36], can compensate for the lack of very long O-antigen chains during growth in the environment of the gall bladder. Nonetheless, our data show that very long O-antigen chains increased the fitness of *S.* Typhimurium in the lumen of the acutely inflamed intestine by mediating bile resistance, which represents the first function ascribed to this surface structure. Inactivation of *yrbK* and *rlpB*, two genes implicated in LPS transport, increases bile resistance of *S.* Typhimurium and correlates with differences in the oligosaccharide units that form long-chain LPS [37]. However, our data suggest that long O-antigen chains do not confer resistance to bile. Conversely, long O-antigen chains confer serum resistance, while very long O-antigen chains are dispensable for this trait [14], [16]. Thus, long O-antigen chains and very long O-antigen chains do not have redundant functions.

The picture emerging from these studies is that very long O-antigen is important for growth of S. Typhimurium in the lumen of the inflamed intestine. Maximum growth in the intestinal lumen is of relevance, because it enhances transmission by the fecal-oral route [22], which is predicted to confer a selective advantage. We conclude that the role of very long O-antigen chains in bile resistance likely represents one of the selective forces that help to maintain the *fepE* gene in the genomes of non-typhoidal *Salmonella* serotypes.

## Materials and Methods

### Bacterial strains, plasmids, and growth conditions

Bacterial strains and plasmids used in this study are presented in Table 2. Cultures of *S.* Typhimurium and *Escherichia coli* were routinely incubated with aeration at 37°C in Luria-Bertani (LB) broth (10 g tryptone, 5 g yeast extract, and 10 g NaCl per liter) or on LB agar plates unless indicated otherwise. Antibiotics were added as necessary at appropriate concentrations: chloramphenicol (Cm), 0.03 mg/ml; carbenicillin (Cb), 0.1 mg/ml; kanamycin (Kan), 0.05 mg/ml; and nalidixic acid (Nal), 0.05 mg/ml.

**Table 2:** Bacterial strains and plasmids used in this study.

Designation	Genotype	Source or Reference
*E. coli* strains		
Top10	F- *mcrA* Δ(*mrr-hsdRMS-mcrBC*) φ80*lacZ*ΔM15 *lacX74 recA1 araD139* Δ(*ara, leu*)7697 *galU galK rpsL endA1 nupG*	Invitrogen
S17-1 λ_pir_	F- *recA thi pro* rK- mK+ RP4:2-Tc::*Mu*Km Tn7 λ_pir_	[40]
*S.* Typhimurium strains		
IR715	ATCC 14028, Nal^R^	[41]
RC31	IR715, *fepE*::pGP704 (Carb^R^)	This study
RC46	IR715, *wzzB*::pEP185.2 (Cm^R^)	This study
SPN452	IR715, *invA*(−9 to +2057)::*tetRA spiB*(+25 to +1209)::KSAC	[9]
RC55	IR715, *invA*(−9 to +2057)::*tetRA spiB*(+25 to +1209)::KSAC *fepE*::pGP704	This study
SW698	IR715, *pmrE*::pFUSE	This study
Plasmids		
pCR2.1	Cloning Vector (Carb^R^, Km^R^)	Invitrogen
pGP704	Suicide plasmid, *oriR6K mobRP4* (Carb^R^)	[42]
pEP185.2	Suicide plasmid, *oriR6K mobRP4* (Cm^R^)	[43]
pFUSE	Suicide plasmid, *oriR6K mobRP4* ‘*lacZYA* (Cm^R^)	[44]
pWSK129	pSC101 *ori* (Km^R^)	[45]
pRC30	pCR2.1::‘*fepE*’	This study
pRC31	pGP704::‘*fepE*’	This study
pRC36	pCR2.1::*fepE*	This study
pRC37	pWSK129::*fepE*	This study
pRC48	pCR2.1::‘*wzzB*’	This study
pRC49	pEP185.2::‘*wzzB*’	This study
pRC54	pSW84::*fepE*	This study
pSW84	pGP704::*phoN*’	[46]
pSW197	pCR2.1::‘*pmrE*’	This study
pSW198	pFUSE::‘*pmrE*’	This study

### Construction of mutants and plasmids

Nucleotide sequences of oligonucleotide primers used for cloning are listed in Table 3. Internal fragment of *S.* Typhimurium the *fepE* and *wzzB* genes were polymerase chain reaction (PCR) amplified using primer pairs 62/63 and 150/151, respectively, and cloned into pCR2.1 (Invitrogen) to give rise to plasmids pRC30 and pRC48. The inserts were excised using restriction endonuclease digestion with *Sma*I and *Sal*I (pRC30) or *Sac*II and *Xho*I (pRC48) and cloned into suicide vector pGP704 digested with *Sma*I and *Sal*I or into suicide vector pEP185.2 digested with *Sac*II and *Xho*I to yield plasmids pRC31 and pRC49, respectively. Plasmids pRC31 and pRC49 were introduced into *S.* Typhimurium IR715 by conjugation with *E. coli* S17-1 λ*pir* and exconjugants were designated RC31 and RC46, respectively. The wild-type *fepE* gene was PCR amplified using the primer pair 52/53 and cloned into pCR2.1 to yield plasmid pRC36. The insert of pCR36 was excised with *Eco*RI and cloned into low copy vector pWSK129 to yield pRC37. To construct a *pmrE* mutant, an internal fragment of the *S.* Typhimurium *pmrE* gene was PCR amplified using primers 263/264, cloned into pCR2.1 to yield plasmid pSW197. The insert of pSW197 was cloned into suicide plasmid pFUSE at *Xba*I and *Sma*I restriction sites to yield plasmid pSW198. Plasmid pSW198 was introduced into *S.* Typhimurium IR715 by conjugation to yield strain SW698. The *fepE*::pGP704 mutation was transduced from RC31 into the *S.* Typhimurium *invA spiB* mutant SPN452 using transduction with phage P22 HT *int*-105 to yield strain RC55.

**Table 3:** Oligonucleotide primers used in this study.

Designation	Purpose	Sequence (5′ to 3′)^a^
50	pGP704 sequencing primer used to confirm insertion mutants	GCATTTATCAGGGTTATTGTCTC
52	Amplification of *fepE* for complementation	TCCCTGCAGCAGGTCATACGGCATGCCATC
53		GGAGAATTCTCAGACTAACCGTTCATCTATC
62	Amplification of *fepE* for insertional inactivation	ATCGCGCCCGGGGGGTTGCTTCTGTCCTTTCTGC
63		TTCAGGGTCGACTTTTACCGCCTGACCATTGC
130	Confirmation of *fepE* insertion	TGCTACCGTTTTCGCCTTTG
131		ACATTCATTGCCGCCAGTTG
150	Amplification of *wzzB* for insertional inactivation	ATCATACCGCGGACAGTTATGGCGTGGGAAGATG
151		CGTCTACTCGAGCGGAGAAAAGACCAGTGGACG
160	Confirmation of *wzzB* insertion	GCTTCATCCTTTTTTTAGTTAGG
161		TTACAAGGCTTTTGGCTTATAGC
170		TTCACCGTCGACGCGAATGGCCTCAATCCG
171		TTTACCTCTAGACAGAGATTCTAACAGCAAGTCAG
172		TTCACCTCTAGATTTACCCGAAAAGCCGGATAGC
173		TTCACGGTCGACCGCATCAGCCGTTATTTACCC
174		TTCACCGTCGACACGGGCGTTGTTTCTGCCTT
175		TTTACCCCCGGGCCCACACTGATGACAAAGC
263	Amplification of *pmrE* for insertional inactivation	TCTAGACCCGCAGCAGTGATG
264		CCCGGGAAAATAAGCGACTCG

a Restriction endonuclease cleavage sites are underlined.

To construct a *fepE* deletion mutant, primers 170 and 171 were used to PCR amplify the region upstream of *S.* Typhimurium *fepE* (flanking region 1) from base 3537 of *entF* to base −48 of *fepE*. Primers 172 and 173 were used to PCR amplify the downstream region of *S.* Typhimurium *fepE* (flanking region 2) from base 1136 of *fepE* to base 322 of *fepC*. PCR products for the *fepE* upstream and downstream regions were gel purified (Qiagen), digested with XbaI, and ligated together with T4 DNA ligase (NEB). The ligation reaction served as a template for PCR amplification of the *fepE* flanking region construct using primers 170 and 173, and this product was gel purified and cloned into pCR2.1 (Invitrogen) to generate pRC50. The cassette containing *fepE* flanking regions was excised from pRC50 using SalI, ligated into SalI-digested pRDH10 using the Quick T4 DNA Ligase kit (NEB) to generate pRC51. The KSAC cassette from pBS34 was excised by XbaI digestion and ligated into the XbaI site of pRC51 to yield pRC52. Plasmid pRC52 was conjugated into *S.* Typhimurium IR715. An exconjugant selected on plates containing LB+Nal+Km was designated RC61 and deletion of *fepE* was confirmed by PCR. For complementation, the *S.* Typhimurium wild type *fepE* allele was PCR amplified using primers 174 and 175 from base −350 of *fepE* to base 1136 of *fepE*, and cloned into pCR2.1 to give rise to plasmid pRC53. The insert was excised using restriction endonuclease digestion with *SalI* and *SmaI* and ligated into *SalI*- and *SmaI*-digested pSW84, a suicide plasmid carrying a DNA fragment upstream of the *phoN* open reading frame, to yield pRC54. Plasmid pRC54 was introduced into RC61 by conjugation with *E. coli* S17-1 λ*pir* and an exconjugant carrying pRC54 integrated into the *phoN* locus was designated RC62.

### Analysis of LPS

Bacterial cultures grown 16 hours at 37°C in LB broth were suspended in 1 ml Dulbecco's phosphate buffered saline (DPBS, Gibco) at a concentration of approximately 1×10^9^ CFU/ml. Samples were washed three times in DPBS (12,000× *g* for 4 minutes). The pellet was resuspended in 0.1 ml lysis buffer (0.5 M Tris-HCl at pH 6.8, 4% sodium dodecyl sulfate [SDS], 40% glycerol, 0.2% bromophenol blue, and 10% β-mercaptoethanol) and boiled for 10 minutes. We then added 0.1 ml of proteinase K solution (Invitrogen) (20 mg proteinase K/ml in 10 mM Tris-HCl, 50% glycerol, and 20 mM CaCl_2_) and incubated for 3 hours at 65°C. LPS preparations were boiled again for 10 minutes, and 0.02 ml of each sample loaded on a 15% SDS-polyacrylamide gel electrophoresis (SDS-PAGE), and visualized by silver staining as described previously [38].

LPS purified from colon and cecal contents was probed with anti-*Salmonella* O:4 serum (Becton, Dickinson and Company) for production of the somatic O antigen by Western blot analysis. CFU of *S.* Typhimurium were determined for each sample and samples loaded in each lane were normalized to represent equivalent amounts of bacteria. Preparations were separated by 15% SDS-PAGE, transferred to an Immobilon PVDF membrane (Millipore) using a Trans-Blot semidry transfer apparatus (Bio-Rad), and detected with anti-*Salmonella* O:4 serum and an alkaline phosphatase-conjugated goat anti-rabbit secondary antibody (Bio-Rad Laboratories). Bands were visualized following exposure and development on a BioSpectrum AC Imaging System (Ultra-Violet Products).

### Determination of MIC values

To determine MIC values of *S.* Typhimurium strains against polymyxin B (Sigma Aldrich), SDS (USB Corporation), sodium choleate (a crude ox bile extract from Sigma Aldrich, which contains the sodium salts of taurocholic, glycocholic, deoxycholic, and cholic acids), or with a combination of sodium choleate and cholestyramine resin (DOW Chemical Company), overnight cultures were washed twice in DPBS, diluted to 1×10^3^ cells per ml with antimicrobial compounds in sterile LB broth (polymyxin B: 0–1.5 µg/ml, increments of 0.1 µg/ml; SDS and sodium choleate: 0–20% w/v, increments of 2%) and incubated for 15 hours at 37°C with aeration to determine the lowest concentration of each antimicrobial compound that prevented bacterial growth. Each experiment was repeated three times independently.

### Serum sensitivity

For analyses of serum sensitivity, 1×10^7^ bacteria from cultures grown to stationary phase were washed three times in DPBS and incubated in 10% human serum for one hour at 37°C with slight agitation. The number of viable bacteria was quantified by spreading serial 10-fold dilutions on LB agar plates containing the appropriate antibiotics.

### Animal experiments

For competitive infection experiments, groups (n = 4) of female mice (C57BL/6J or 129/svJ Jackson Laboratory) aged 6 to 8 weeks were inoculated intragastrically with 20 mg streptomycin in a volume of 0.2 ml 48 hours prior to intragastric inoculation with either 0.1 ml sterile LB broth or 1×10^7^ CFU of the indicated mixtures of *S.* Typhimurium strains. Alternatively, animals were inoculated intragastrically with either 0.1 ml sterile LB broth or 1×10^7^ CFU of the indicated mixtures of *S.* Typhimurium strains without being pretreated with streptomycin. Fecal pellets were collected and organs were harvested at day 4 (C57BL/6J) or day 7 (129/svJ) after infection. Fecal and tissue samples were homogenized in PBS, serially diluted and plated on selective media to enumerate CFU. The competitive index (CI) was calculated by dividing the ratio of wild-type to mutant bacteria in homogenates (output) by the ration present in the inoculum (input). In some experiments, 129/SvJ mice were fed a normal rodent diet (Harlan-Teklad) supplemented with the bile acid sequestrant cholestyramine resin (2%, The Dow Chemical Company) starting the day before streptomycin treatment and continuing throughout the experiment.

For metabolite profiling, groups of 4 mice (C57BL/6J) received 20 mg streptomycin in a 0.1 ml volume intragastrically and were inoculated intragastrically 24 hours later with either 0.1 ml sterile LB broth or 1×10^9^ CFU of *S.* Typhimurium in LB broth. The cecum was collected four days after infection, contents were removed and metabolites from the mucosal surface were extracted by five washes with 0.2 ml sterile UltraPure distilled water (Gibco). Debris was removed by centrifugation at 20,000× *g* for 5 minutes, the supernatant was filter sterilized to remove bacteria and submitted to metabolite profiling analysis.

### Ethics statement

All animal experiments were performed according to Association for Assessment and Accreditation of Laboratory Animal Care (AAALAC) guidelines. All animal experiments were approved by the Institutional Animal Care and Use Committee at the University of California, Davis.

### Histopathology

Cecal tissues were formalin fixed, sectioned, stained with hematoxylin and eosin (H&E), and submitted to a veterinary pathologist for blinded scoring using a scale described previously [8]. Representative images of tissue sections were taken using an Olympus BX41 microscope.

### Metabolite profiling

Reagents and standards: Liquid chromatography/mass spectrometry (LC/MS) grade acetonitrile and acetone (Burdick and Jackson, VWR International, West Chester, PA, USA) and extra pure ammonium formate (EMD, Gibbstown, NJ, USA) were purchased. Ultrapure water was supplied by an in house Millipore system (Billerica, MA, USA). Each lot of organic solvents was investigated by LC/MS infusion. Utilizing ultrapure water, aqueous buffer for liquid chromatography/electrospray ionization mass spectrometry (LC/ESI-MS) and a stock solution (1 mg/ml) of tuning reference compound was prepared freshly on a daily basis in a solvent system identical to the initial mobile phase composition of LC.

Hydrophilic interaction chromatography (HILIC)-LC/ESI-MS profiling: HILIC-LC/ESI-MS analysis [39] was performed with the use of a modified silica-based column (Luna HILIC Diol, 150_3 mm, 3 mm particle size; Phenomenex, Torrance, CA, USA). The mobile phases were 100 mM ammonium formate (pH 4.0) (A) and acetonitrile (B) (flow rate 0.4 mL/min at 40°C). After a 2 minute isocratic run at 3% A, a sequential ramping scheme was followed up to 40% A for total injection time of 20 min. The injection volume was set to 10 ml. The entire effluent from the high-pressure liquid chromatography (HPLC) column was directed into the ESI source of a linear trap quadrupole (LTQ) linear ion trap (LIT) mass spectrometer (Thermo Fisher, San Jose, CA, USA) operated under Xcalibur software (v1.4, Thermo Fisher). The electrospray voltage was set to 5 kV. Nitrogen sheath and auxiliary gas flow were set at 60 and 20 arbitrary units, respectively. The ion transfer capillary temperature was set at 350°C with typical ion gauge pressure of 0.90×10^−5^. Full scan spectra were acquired from 100–1500 amu at unit mass resolution with maximum injection time set to 200 ms in one micro scan. Acquisition was performed in both positive/negative switching modes. A sucrose tune file in negative/positive modes at high LC flow rate was used during all the LC/ESI-MS acquisitions on the LTQ mass spectrometer.

Metabolite annotation: Annotation of the MS and MS/MS spectra was done using commercial NIST05/Wiley Registry; METLIN, Mass-Bank, Human Metabolome Database, Lipid Maps, and in-house built mass spectral libraries. Annotation was further validated with de novo structure identification. The unique elemental formula was searched against the CAS database using the strategy of Explore Substances – Chemical Structure (American Chemical Society, Washington, DC) for known compounds, or input into the MolGen 3.5,20 generating all of the possible structural isomers corresponding to the elemental formula. The chemical structures were saved and imported to Mass Frontier 5.0 (HighChem Ltd., Bratislava, Slovakia) for MS/MS fragmentation modeling analysis. The Mass Frontier Fragments and Mechanisms module is an expert system providing information about basic fragmentation and rearrangement processes based on literature, starting from a user-supplied chemical structure. The theoretical fragments generated by Mass Frontier were compared to those acquired from LC/MS. Parent compounds that had the best match of MS/MS fragmentation pattern were considered as the molecular structures of the potential biomarkers. For validation purpose, the proposed molecules were searched against chemical structure and property databases or search engines such as publicly available PubChem, Chemical Structure Lookup Service (CSLS), CRC Dictionary of Natural Products (DNP), ChemSpider, and proprietary Beilstein Database using MDL Crossfire Commander and Chemical Abstracts Database (CAS) using SciFinder Scholar.

Raw data processing: Prior to data processing, original Xcalibur LC/MS files were converted into netCDF format using the XConverter (Thermo Fisher), then converted into WIFF format usingAnalyst QS for use in MarkerView software (v1.2, Applied Biosystems, Foster City, CA, USA). For HILIC LC/MS data, peak finding options were set as follows: subtraction offset, 10 scans; subtraction multiplication factor, 1.3; noise threshold, 3; minimum spectral peak width, 5 amu; minimum retention time peak width, 5 scans; and maximum retention time width, 1000 scans. Peak alignment options were set as follows: retention time tolerance, 0.5 min; mass tolerance, 0.8 amu; and maximum number of peaks, 5000. Peaks found in fewer than 3 of the samples were discarded using filter setting. Peak area integration was performed using raw data. Datasets were normalized using Euclidean norm by scaling each sample-vector to unit vector norm, which can be interpreted geometrically as a projection of the samples x to a hyper-sphere with the length of this sample vector scaled to one. Peaks were, then, normalized to the total absolute area of all detected metabolites in each sample. Datasets were routinely processed in MarkerView for principal component analysis. The data that constituted retention time, mass-to-charge ratio, and peak areas of detected and aligned peaks were exported from MarkerView into into MetaboAnalyst and MetPA for further analysis.

### Quantitative determination of total bile acids

Colon contents were weight, homogenized in 1 ml PBS and analyzed using a colorimetric Total Bile Acids Assay Kit (Bio-Quant). Briefly, conversion of bile acids and Thio-NADH to 3-keto steroids and Thio-NADH by 3-α.hydroxysteroid dehydrogenase was followed by measuring change of absorbance at 405 nm. Bile acid concentrations were determined using a conjugated cholic acids standard using a protocol provided by the manufacturer.

### Statistical analysis

Competitive indices were converted logarithmically (log_10_) prior to statistical analysis using a one-tailed parametric test (Student's *t* tests). Concentrations of total bile acids were analyzed using a one-tailed Student's *t* tests. A *P* value of <0.05 was considered to be significant.

Fold differences in metabolites between mock-infected mice and *S.* Typhimurium infected mice were converted logarithmically (log_2_) and a Student's *t* was used to determine whether differences were statistically significant. False discovery rates were estimated using the *q* value method. A *P* value of <0.05 and *q* value of <0.1 were considered a significant change.

## Supporting Information

Figure S1 Very long O-antigen chains are not required for serum resistance. (A) Silver stained SDS-PAGE of LPS preparations from the indicated *S.* Typhimurium strains. Plasmid pRC37 carries the cloned *fepE* gene. Positions of short, long and very long O-antigen chains (arrows) are indicated on the right. (B) Recovery of the indicated *S.* Typhimurium strains after one-hour incubation in 10% human serum. ns, not significantly different.
(PDF)

Figure S2 Very long O-antigen chains confer a fitness advantage in the C57BL/6 mouse colitis model. (A and B) C57BL/6J mice were pre-treated with streptomycin and infected with an equal mixture of the *S.* Typhimurium wild type and a *fepE* mutant. (A) Competitive indices recovered from feces and colon contents. (B) Competitive indices recovered from the liver and spleen. Bars represent geometric means ± standard error.
(PDF)

Figure S3 Very long O-antigen chains are expressed *in vivo*. Detection of very long O-antigen (VL Oag) by Western blot in samples from mice used in the experiment depicted in Figure 1. Mice (129/SvJ) were treated with streptomycin (strep+) or were left untreated (strep−) and were subsequently infected with sterile medium (LB control, lanes 7 and 8)) or with an equal mixture of the *S.* Typhimurium wild type and a *fepE* mutant (WT/*fepE*, lanes 3–6). Samples from *in vitro* grown *S.* Typhimurium wild type (lane 1) and a *fepE* mutant (lane 2) were loaded as a control. The amount of LPS loaded in each lane was normalized based on CFU counts for *S.* Typhimurium. NA, not applicable.
(PDF)

Figure S4 Very long O-antigen chains confer a fitness advantage in the CBA/J mouse colitis model. (A and B) CBA/J mice were pre-treated with streptomycin and infected with an equal mixture of the *S.* Typhimurium wild type (wt) and a *fepE* mutant (competitive infection design). (A) Competitive indices (CI) recovered from feces and colon contents. (B) Competitive indices recovered from the liver and spleen. (C) CBA/J mice were pre-treated with streptomycin and infected with an equal mixture of a *S.* Typhimurium *fepE* mutant (RC61) and a derivative of RC61 carrying an intact copy of the *fepE* gene inserted chromosomally (RC62). Competitive indices recovered from feces, colon contents, liver or spleen at the indicated time points are shown. (D and E) Mice (CBA/J) were pre-treated with streptomycin and infected with either the *S.* Typhimurium wild type (open bars) or a *fepA* mutant (closed bars) (single infection design). (D) CFU recovered from the liver and spleen. (E) CFU recovered from colon contents or from feces over time. Bars represent geometric means ± standard error. **, *P*<0.01, ns, not significantly different.
(PDF)

Figure S5 Principle component analysis score plot of HILIC-LC/ESI-MS data. Principle component analysis of data constituting retention time, mass-to-charge ratio, and peak areas of detected and aligned peaks was performed for samples from four mock-infected mice (63_1, 63_2, 63_3 and 63_4) and four *S.* Typhimurium-infected mice (64_1, 64_2, 64_3 and 64_4). Dashed lines in the principle component analysis plot illustrate that samples from *S.* Typhimurium-infected mice (green crosses) were well separated from mock-infected controls (red triangles).
(PDF)

Figure S6 Very long O antigen chains confer a fitness advantage at concentrations of bile acids that are below the MIC of a *fepE* mutant. The *S.* Typhimurium wild type (IR715) or a *fepA* mutant (RC31) were grown in LB broth containing 3% (A), 0.3% (B), 0.03% (C) or 0.003% sodium choleate (D) and growth was followed by measuring the optical density at a wavelength of 600 nm (OD_600_).
(PDF)

Table S1 Compounds identified by metabolite profiling. m/z_Rt (min), mass to charge ratio (m/z) and retention time (Rt) in minutes.
(PDF)
