# Supplementary figures and images for: Very Long O-antigen Chains Enhance Fitness during Salmonella-induced Colitis by Increasing Bile Resistance
Source: PLoS Pathog. 2012 Sep 20;8(9):e1002918. doi: 10.1371/journal.ppat.1002918 (PMC3447750; doi:10.1371/journal.ppat.1002918)

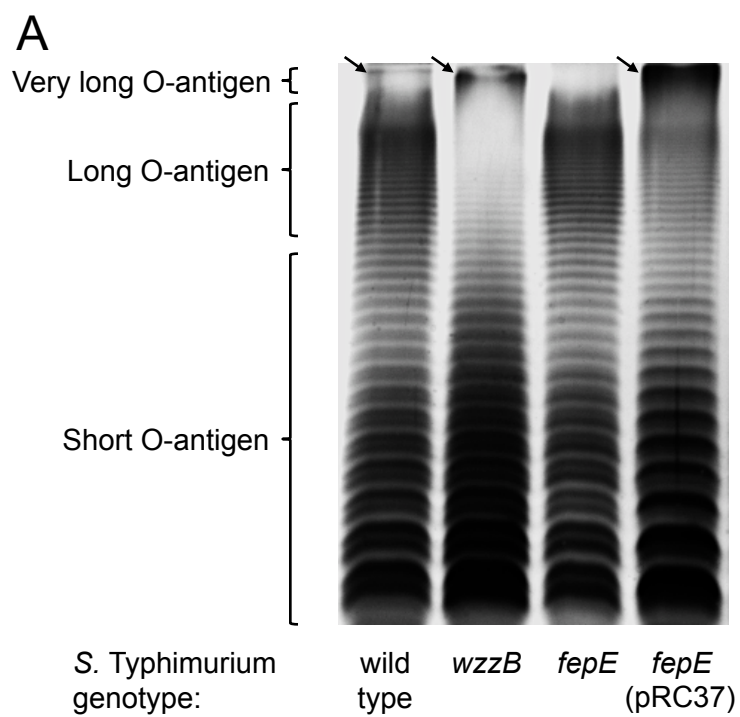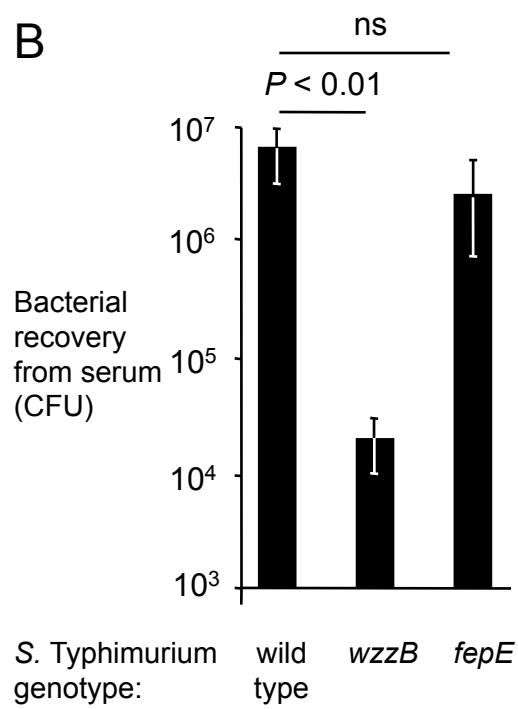

Supplement: Figure S1 — Very long O-antigen chains are not required for serum resistance. (A) Silver stained SDS-PAGE of LPS preparations from the indicated S. Typhimurium strains. Plasmid pRC37 carries the cloned fepE gene. Positions of short, long and very long O-antigen chains (arrows) are indicated on the right. (B) Recovery of the indicated S. Typhimurium strains after one-hour incubation in 10% human serum. ns, not significantly different. (PDF) [file ppat.1002918.s001.pdf]

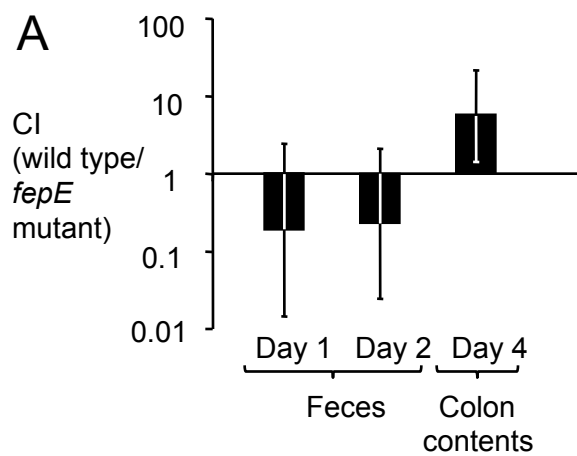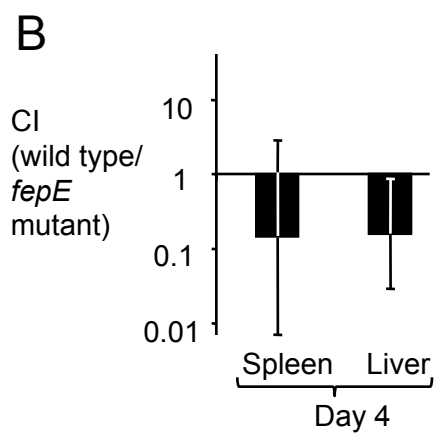

Supplement: Figure S2 — Very long O-antigen chains confer a fitness advantage in the C57BL/6 mouse colitis model. (A and B) C57BL/6J mice were pre-treated with streptomycin and infected with an equal mixture of the S. Typhimurium wild type and a fepE mutant. (A) Competitive indices recovered from feces and colon contents. (B) Competitive indices recovered from the liver and spleen. Bars represent geometric means ± standard error. (PDF) [file ppat.1002918.s002.pdf]

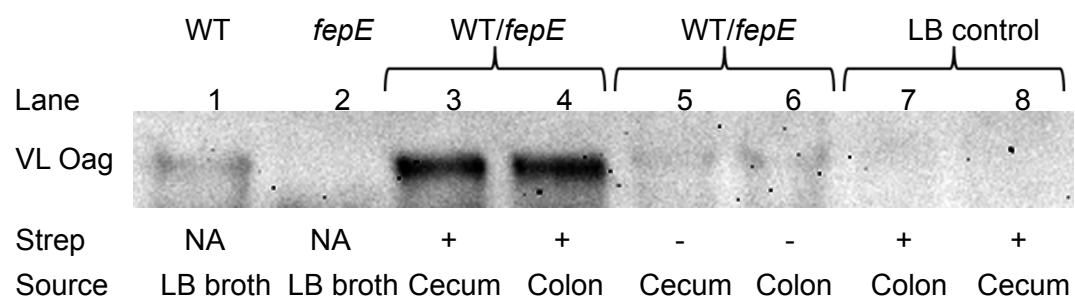

Supplement: Figure S3 — Very long O-antigen chains are expressed in vivo. Detection of very long O-antigen (VL Oag) by Western blot in samples from mice used in the experiment depicted in Figure 1. Mice (129/SvJ) were treated with streptomycin (strep+) or were left untreated (strep−) and were subsequently infected with sterile medium (LB control, lanes 7 and 8)) or with an equal mixture of the S. Typhimurium wild type and a fepE mutant (WT/fepE, lanes 3–6). Samples from in vitro grown S. Typhimurium wild type (lane 1) and a fepE mutant (lane 2) were loaded as a control. The amount of LPS loaded in each lane was normalized based on CFU counts for S. Typhimurium. NA, not applicable. (PDF) [file ppat.1002918.s003.pdf]

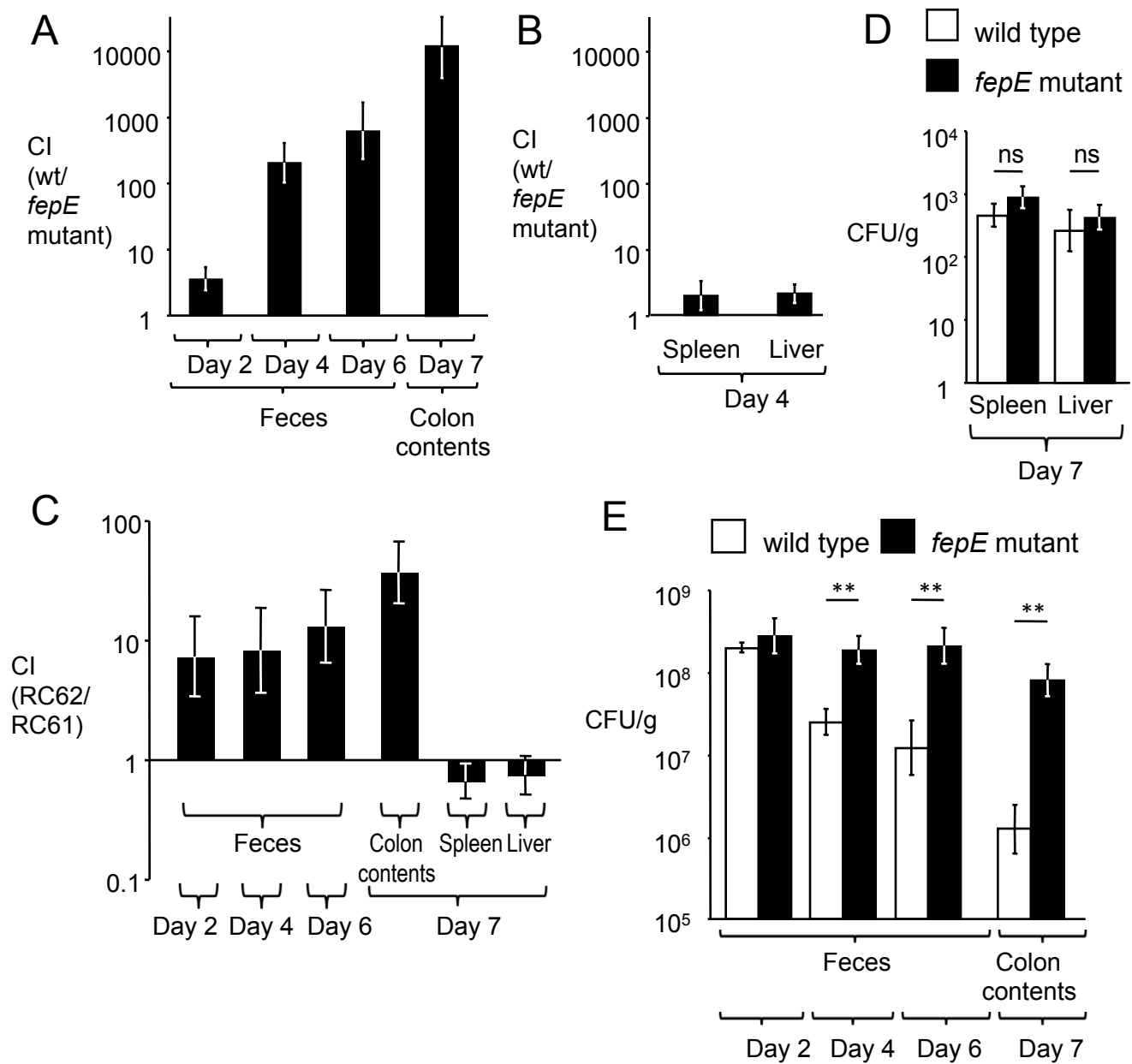

Supplement: Figure S4 — Very long O-antigen chains confer a fitness advantage in the CBA/J mouse colitis model. (A and B) CBA/J mice were pre-treated with streptomycin and infected with an equal mixture of the S. Typhimurium wild type (wt) and a fepE mutant (competitive infection design). (A) Competitive indices (CI) recovered from feces and colon contents. (B) Competitive indices recovered from the liver and spleen. (C) CBA/J mice were pre-treated with streptomycin and infected with an equal mixture of a S. Typhimurium fepE mutant (RC61) and a derivative of RC61 carrying an intact copy of the fepE gene inserted chromosomally (RC62). Competitive indices recovered from feces, colon contents, liver or spleen at the indicated time points are shown. (D and E) Mice (CBA/J) were pre-treated with streptomycin and infected with either the S. Typhimurium wild type (open bars) or a fepA mutant (closed bars) (single infection design). (D) CFU recovered from the liver and spleen. (E) CFU recovered from colon contents or from feces over time. Bars represent geometric means ± standard error. **, P<0.01, ns, not significantly different. (PDF) [file ppat.1002918.s004.pdf]

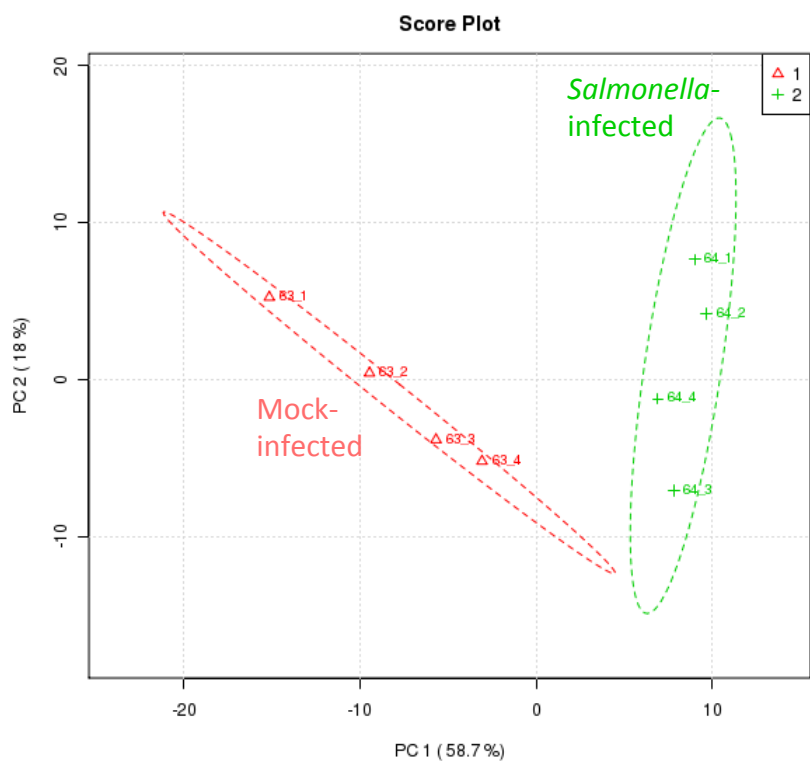

Supplement: Figure S5 — Principle component analysis score plot of HILIC-LC/ESI-MS data. Principle component analysis of data constituting retention time, mass-to-charge ratio, and peak areas of detected and aligned peaks was performed for samples from four mock-infected mice (63_1, 63_2, 63_3 and 63_4) and four S. Typhimurium-infected mice (64_1, 64_2, 64_3 and 64_4). Dashed lines in the principle component analysis plot illustrate that samples from S. Typhimurium-infected mice (green crosses) were well separated from mock-infected controls (red triangles). (PDF) [file ppat.1002918.s005.pdf]

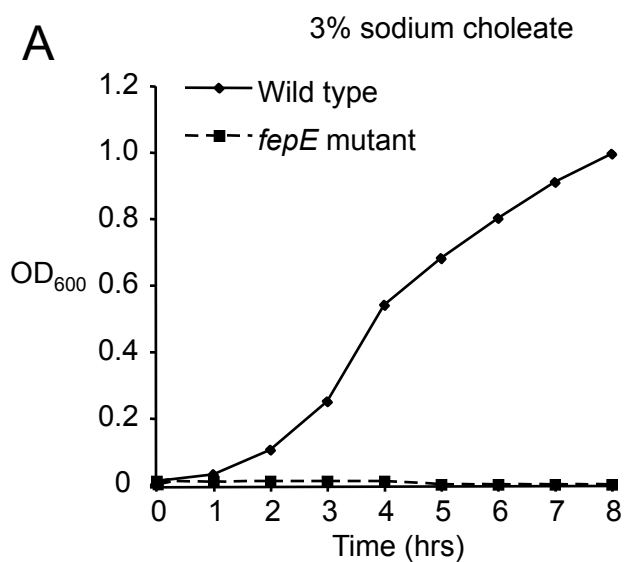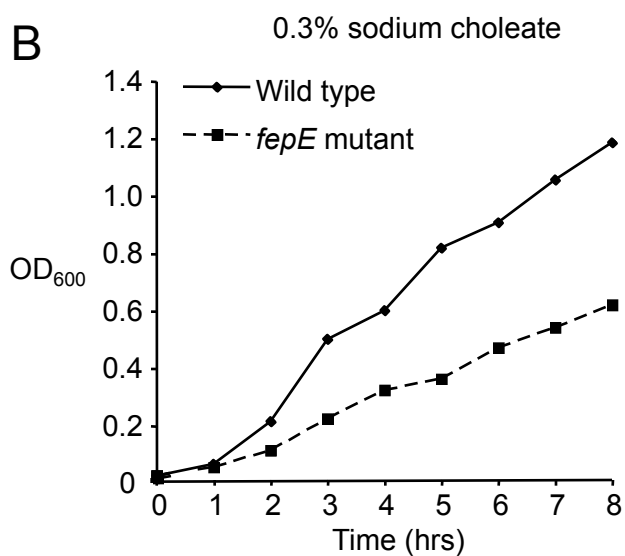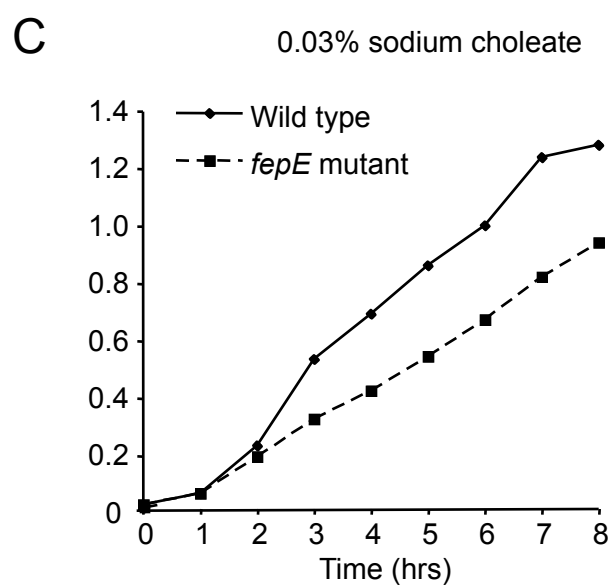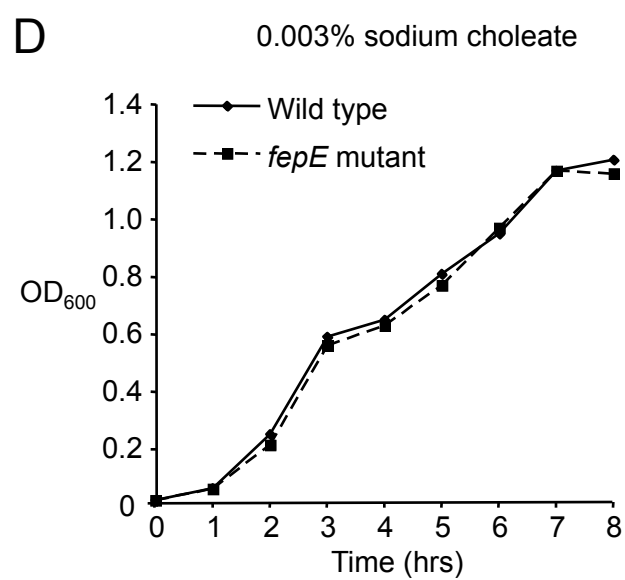

Supplement: Figure S6 — Very long O antigen chains confer a fitness advantage at concentrations of bile acids that are below the MIC of a fepE mutant. The S. Typhimurium wild type (IR715) or a fepA mutant (RC31) were grown in LB broth containing 3% (A), 0.3% (B), 0.03% (C) or 0.003% sodium choleate (D) and growth was followed by measuring the optical density at a wavelength of 600 nm (OD600). (PDF) [file ppat.1002918.s006.pdf]
