# Supplementary material for: Very Long O-antigen Chains Enhance Fitness during Salmonella-induced Colitis by Increasing Bile Resistance
Source: PLoS Pathog. 2012 Sep 20;8(9):e1002918. doi: 10.1371/journal.ppat.1002918 (PMC3447750; doi:10.1371/journal.ppat.1002918)
Supplement: Table S1 — Compounds identified by metabolite profiling. m/z_Rt (min), mass to charge ratio (m/z) and retention time (Rt) in minutes. (PDF) [file ppat.1002918.s007.pdf]

Table S1: Metabolites identified by metabolite profiling

Index	Substance Name	m/z_Rt (min)	t-value	p-value	Mean 1	Mean 2	Median 1	Median 2	Sigma 1	Sigma 2	Delta	Fold Change	Log (Fold Change)
1	Malic acid	135.3067_7.24	3.706414887	0.01001	16751000	10853250	15723000	10852000	2941275.1	1215287.2	5897750	1.543408656	0.188480932
2	Proline betaine	144.3610_10.75	0.524086876	0.61901	320500	298750	323500	271000	39196.088	73163.629	21750	1.072803347	0.03052012
3	3-Methylglutaconic acid	145.2167_13.58	-0.74047337	0.48697	1737500	2170000	1710000	1870000	268498.91	1136896.4	-432500	0.800691244	-0.09653492
4	3-Dehydroxycarnitine	146.2290_23.75	-0.74581164	0.48396	393500	431250	384500	455500	79181.227	63073.37	-37750	0.912463768	-0.03978437
5	Isobutyrylglycine	146.2625_8.52	3.07838825	0.02171	1539750	862086.75	1420500	818805.5	424217.22	117807.94	677663.25	1.786073153	0.251899243
6	4-Acetamidobutyric acid	146.5922_5.86	4.514609146	0.00404	1247500	639750	1210000	616000	249582.99	100979.78	607750	1.949980461	0.29003026
7	Acetylcholine	147.0648_17.67	2.692618001	0.03593	4877500	3432500	4930000	3400000	944964.73	508944.99	1445000	1.420975965	0.152586732
8	l-Lysine	147.1803_21.15	3.674802632	0.01039	2402500	1433500	2442500	1482500	378141.51	367605.31	969000	1.675967911	0.224265699
9	l-Glutamine	147.3541_12.24	2.299753	0.06112	4937500	2800000	5160000	2735000	654083.33	1740019.2	2137500	1.763392857	0.246349077
10	l-Glutamic acid	148.1069_19.63	6.166226432	0.00084	6215000	3307500	6455000	3405000	628039.28	703485.37	2907500	1.879062736	0.27394128
11	Mevalonic acid	149.2792_12.27	1.503383378	0.18343	1970000	1229500	2040000	1266500	564387.57	807410.06	740500	1.602277349	0.204737693
12	Acetylhistamine	154.1890_8.01	4.104611694	0.00632	4837500	3525000	4925000	3640000	564350.66	300832.18	1312500	1.372340426	0.137461857
13	Valeryl-glycine	160.1932_6.53	-1.45043646	0.19713	57604750	64523250	55672000	64925000	7654702.4	5693415	-6918500	0.892775085	-0.04925794
14	Homomethionine	162.2119_12.29	3.076006813	0.02177	50662500	30712500	47865000	30000000	7316494.5	10710983	19950000	1.64957265	0.217371447
15	Phenylalanine	166.1484_14.54	3.382302659	0.01482	21690000	9862500	20430000	9335000	6574790.2	2384273.1	11827500	2.199239544	0.342272536
16	Arginine	175.0923_23.07	-1.33584831	0.23003	7351250	8781000	7174000	8878500	1004346	1890344.8	-1429750	0.837176859	-0.07718278
17	Ferulic acid	178.2971_6.96	-0.59269984	0.57502	9628750	10238750	9644500	9679000	677649.55	1943632.9	-610000	0.940422415	-0.02667703
18	L-tyrosine	180.3420_8.54	3.380749339	0.01484	5966250	4574000	6190000	4541500	638061.84	520816.67	1392250	1.304383472	0.115405287
19	Pyridoxic Acid	184.0768_4.74	3.714504881	0.00992	12209750	8997500	12044500	8809500	1343535	1089187.6	3212250	1.357015838	0.132584916
20	Phopshocholine	184.1973_27.03	-0.04320014	0.96694	1727500	1732500	1665000	1695000	159033.54	168201.27	-5000	0.997113997	-0.00125519
21	Acetylmethionine	192.1621_2.90	-0.18503372	0.8593	6362500	6460000	6510000	6260000	929852.14	495983.87	-97500	0.984907121	-0.00660472
22	Erythrose-4-phosphate	199.4013_17.61	0.44362806	0.67286	7215000	6987500	7280000	6875000	806205.1	634001.84	227500	1.03255814	0.013914515
23	Lauric acid	201.1686_1.94	-1.89453672	0.10698	9257500	10272500	9285000	10300000	895037.24	589088.28	-1015000	0.901192504	-0.04518243
24	3-Indolylbutyric acid	204.0969_8.08	-0.33455891	0.74934	80781847	84344500	82330500	76631000	6055741.4	20418530	-3562653	0.957760693	-0.01874299
25	N-Acetylcarnitine	204.1603_14.88	1.269675604	0.25122	797500	541500	616500	546500	397665.27	66895.441	256000	1.472760849	0.168132231
26	Phenylpropionylglycine	207.1820_10.26	-1.12043294	0.30536	4774000	5241500	4754000	5046500	445219.05	705810.41	-467500	0.910807975	-0.04057318
27	butyrylcarnitine	232.1476_13.36	1.804687631	0.12116	25380000	22207500	24305000	22460000	3011323.1	1814687.8	3172500	1.142857143	0.057991947
28	Deoxyadenosine	252.1070_5.36	0.292538291	0.77973	6249750	6155000	6224000	6070000	274355.46	586810.02	94750	1.015393989	0.006634588
29	Nicotinamide riboside	254.4716_10.11	1.699053	0.14022	22175250	17643250	22076500	15991000	1883085.3	4991332.8	4532000	1.256868774	0.099289937
30	Adenosine	267.2000_7.61	4.100296906	0.00636	55426750	43911750	54260000	43268500	4312610.8	3598378.8	11515000	1.262230496	0.101138669
31	unknown phosphate	287.9971_13.79	-3.21425142	0.01827	77856.75	195000	81149	173000	8618.1464	72378.634	-117143.3	0.399265385	-0.39873834
32	unknown phosphate	289.0093_14.51	-1.84741441	0.1142	4792500	6777500	4835000	6645000	595671.89	2064741.7	-1985000	0.707119144	-0.1505074
33	fatty acid	290.1145_4.19	3.891381132	0.00806	#########	78969750	#########	79198500	13335587	3544888.4	26848000	1.339978283	0.12709776
34	Phe-Hydroxy-Proline	295.0237_12.37	-0.63852822	0.54671	#########	#########	#########	#########	23544445	38513057	-14411500	0.935117309	-0.0291339
35	unknown phosphate	296.7859_15.94	-4.91099187	0.00268	92047750	#########	94544500	#########	11699498	22422484	-62102500	0.597130073	-0.22393106
36	GlcNAc 1-phosphate	302.1563_19.66	2.41098856	0.0525	#########	#########	#########	#########	16442412	12007904	24544250	1.151199018	0.06115041
37	cTMP	304.0685_23.77	-1.81340764	0.11971	18960000	22952500	18770000	22610000	2022177	3911516.1	-3992500	0.826053807	-0.08299166
38	2',3'-cUMP	305.2156_7.18	-0.68080135	0.52139	#########	#########	#########	#########	9205626.4	4823774.5	-3537750	0.970001336	-0.01322767
39	heneicosanoic acid	325.2477_2.63	4.578673124	0.00378	65415000	54140000	65125000	54465000	2126099.7	4442454.3	11275000	1.208256372	0.082159094
40	cAMP	329.1749_23.78	-1.5864308	0.16374	9626500	10747500	9770000	10365000	584016.84	1286918.2	-1121000	0.895696674	-0.04783904
41	eicosanoid	377.2309_13.58	-3.48538802	0.01306	66310000	#########	70305000	#########	18136598	18412767	-45040000	0.595509654	-0.22511119
42	7-ketolithocholic acid	391.2419_2.65	4.685845376	0.00338	7942500	3138250	7250000	3191000	2040904.6	198521.83	4804250	2.530869115	0.403269686
43	cholic acid	407.2666_4.13	1.842211446	0.11503	#########	#########	#########	#########	#########	43614785	#########	1.122722011	0.050272237
44	LysoPC (10:0)	434.1167_14.26	-3.87080596	0.00826	67350000	74950000	67050000	74700000	3512359.1	1755942.3	-7600000	0.898599066	-0.04643404
45	Lyso-PE(14:1)	462.1960_16.05	-2.60634703	0.04032	3140000	4832500	3185000	4590000	129871.73	1292242.9	-1692500	0.649767201	-0.18724221
46	C31:0	465.3139_2.35	0.113725732	0.91317	36702500	36080000	32565000	36950000	10266568	3800385.9	622500	1.017253326	0.007429118
47	LysoPC(14:0)	466.0299_3.87	0.890660819	0.4074	15575000	12905000	16050000	10550000	2830047.1	5285587.3	2670000	1.206896552	0.081670046
48	dCTP	467.1937_19.74	2.794244385	0.0314	7782500	5412500	7255000	5495000	1666100.7	318891.31	2370000	1.437875289	0.15772122
49	Triacylglycerol	468.0147_13.12	-1.84061604	0.11528	3495000	4312500	3565000	4250000	777281.59	429990.31	-817500	0.810434783	-0.09128193
50	Phosphatidylinositol 3-phosphate	471.0988_23.65	-5.58993724	0.00139	32221500	41587000	32052000	41478500	806420.28	3252358	-9365500	0.774797413	-0.11081184

51	LysoPC(16:1)	480.1061_15.95	-3.1776994	0.01913	6383950	15706075	6438000	14705950	240035.45	5862304.2	-9322125	0.406463741	-0.39097819
52	Cer(28:0)	483.2523_19.32	4.228582936	0.00551	5607500	4537500	5565000	4515000	478147.47	165806.11	1070000	1.235812672	0.091952644
53	7-Sulfocholate	487.2914_13.05	-2.48109834	0.04773	16225000	21800000	17050000	20950000	3796818.8	2404163.1	-5575000	0.744266055	-0.12827179
54	PA(22:2)	491.1135_17.49	3.795845024	0.00901	#########	#########	#########	#########	14868422	4388259.9	29422500	1.269134899	0.103507787
55	LysoPC(16:1)	492.3034_21.67	1.417105378	0.20622	3570000	3295000	3605000	3335000	228764.8	313528.31	275000	1.083459788	0.034812797
56	LysoPC (16:0)	496.1184_15.97	-3.32799903	0.01585	331750	1202250	343500	1146500	50835.519	520661.36	-870500	0.275940944	-0.55918385
57	Tauromuricholic acid	514.2225_16.02	-3.54740218	0.01211	2768500	8705000	2854000	7905000	328353.98	3330810.7	-5936500	0.318035612	-0.49752425
58	Taurocholic acid	514.2983_12.78	-0.72524535	0.4956	9953250	10931250	10039500	11288500	2152648.8	1624811	-978000	0.910531732	-0.04070491
59	DG(16:0/16:0/0:0)	567.1988_6.86	-1.58441976	0.16419	#########	#########	#########	#########	17194149	24265684	-23560250	0.834403735	-0.07862376
60	LysoPC(22:0)	570.3170_22.69	1.270977819	0.25079	2922500	2730000	2945000	2785000	112952.79	281069.39	192500	1.070512821	0.029591873
61	Urobilin	595.0470_3.22	3.041967247	0.02275	82475000	56827500	81100000	53070000	12804784	10971759	25647500	1.451321983	0.161763774
62	DG(37:2)	635.0508_15.34	-6.1802387	0.00083	#########	#########	#########	#########	13333458	10126350	-51737500	0.715704591	-0.1452662
63	PG(28:0)	665.1509_18.14	1.814941695	0.11945	12180000	10745000	12245000	10870000	617899.67	1455598.4	1435000	1.133550489	0.054440868
64	PC(32:5)	722.1592_17.42	6.133942015	0.00086	8955000	6415250	8955000	6293500	699261.52	443596.95	2539750	1.3958926	0.144852005
65	PC(38:4)	811.1321_16.76	1.244848767	0.2596	42927500	40592500	43060000	39455000	2078178.3	3123239.5	2335000	1.057522941	0.024289798
66	PI(36:4)	857.5153_17.81	21.27804635	7.03E-07	34587575	22814900	34715500	22765750	695082.69	861003.05	11772675	1.516008179	0.180701544
67	LacCer(d18:1/18:1)	886.2899_18.88	11.91742123	2.11E-05	6092500	3300000	6275000	3290000	450360.97	129614.81	2792500	1.846212121	0.266281598
